# The Sugar Transporter MST1 Is Involved in Colonization of Rhizosphere and Rhizoplane by Metarhizium *robertsii*

**DOI:** 10.1128/mSystems.01277-21

**Published:** 2021-12-14

**Authors:** Jin Dai, Wubin Mi, Congcong Wu, Hui Song, Yuting Bao, Mingxiang Zhang, Sufen Zhang, Weiguo Fang

**Affiliations:** a MOE Key Laboratory of Biosystems Homeostasis and Protection, Institute of Microbiology, College of Life Science, Zhejiang University, Hangzhou, China; b College of Agriculture and Biotechnology, Institute of Nuclear-Agricultural Science, Zhejiang University, Hangzhou, China; Oak Ridge National Laboratory

**Keywords:** plant-symbiotic fungi, *Metarhizium*, sugar transporter, rhizosphere colonization, rhizoplane colonization, rhizosphere fungi

## Abstract

It is widely recognized that plant-symbiotic fungi are supported by photosynthates; however, little is known about the molecular mechanisms underlying the utilization of plant-derived sugars by rhizospheric fungi. In the insect-pathogenic and plant-symbiotic fungus Metarhizium robertsii, we previously showed that the utilization of oligosaccharides by the transporter MRT (*Metarhizium* raffinose transporter) is important for rhizosphere competency. In this study, we identified a novel monosaccharide transporter (MST1) that is involved in the colonization of the rhizoplane and acts additively with MRT to colonize the rhizosphere. MST1 is not involved in infection of insects by M. robertsii. MST1 is an H^+^ symporter and is able to transport a broad spectrum of monosaccharides, including glucose, sorbose, mannose, rhamnose, and fructose. Deletion of the *Mst1* gene impaired germination and mycelial growth in medium containing the sugars that it can transport. Homologs of MST1 were widely found in many fungi, including plant symbionts such as *Trichoderma* spp. and mycorrhizal fungi and plant pathogens such as Fusarium spp. This work significantly advances insights into the development of symbiotic relationships between plants and fungi.

**IMPORTANCE** Over 90% of all vascular plant species develop an intimate symbiosis with fungi, which has an enormous impact on terrestrial ecosystems. It is widely recognized that plant-symbiotic fungi are supported by photosynthates, but little is known about the mechanisms for fungi to utilize plant-derived carbon sources. In the fungus Metarhizium robertsii, we identified a novel monosaccharide transporter (MST1) that is an H^+^ symporter and can transport a broad spectrum of monosaccharides, including glucose, sorbose, mannose, rhamnose, and fructose. MST1 is involved in the colonization of the rhizoplane and acts additively with the previously characterized oligosaccharide transporter MRT to colonize the rhizosphere. Homologs of MST1 were found in many fungi, including plant symbionts and plant pathogens, suggesting that the utilization of plant-derived sugars by MST1 homologs could also be important for other fungi to develop a symbiotic or parasitic relationship with their respective plant hosts.

## INTRODUCTION

Over 90% of all vascular plant species develop an intimate symbiosis with fungi, which has an enormous impact on terrestrial ecosystems (1, 2). Plant-derived fatty acids in roots are important carbon sources for fungi to colonize plant tissue (3, 4). Plants also exude a substantial amount (11 to 40%) of photosynthesis-derived carbon into the rhizosphere, which includes sugars, amino acids, organic acids, fatty acids, and secondary metabolites, with sugars usually being the major components (5, 6). Since there is a scarcity of easily utilizable carbon sources in the soil for many microbes, it is generally accepted that plant-derived photosynthates support the microbial population in the rhizosphere. Rhizospheric microbes need to actively compete with each other for the carbon sources exuded by plant roots. Therefore, elucidation of the mechanisms for the acquisition of plant-derived carbon sources is important for understanding microbial ecology in the rhizosphere. However, due to the complexity of root exudates and the complicated mechanisms for fungi to acquire carbon sources from their environments, it is highly difficult to determine the roles of different plant components and their fungal counterparts in these rhizospheric processes (7).

The ascomycete fungus Metarhizium robertsii is an insect pathogen and a plant root symbiont (8). It can colonize the rhizosphere and rhizoplane of diverse plant species, and it is also able to grow endophytically in some plant species (9). The mutualistic interactions offer benefits to the fungus and plants. M. robertsii can improve plant growth, development, and health as it is antagonistic to plant pathogens and herbivores and can transfer insect-derived nitrogen to the plants and enhance plant tolerance to abiotic stresses (10). The biochemical and genetic mechanisms underlying infection of insects by *M. robertsii* are generally well established. However, the study of how this fungus develops symbiotic relationships with plant roots is still in its infancy. In fact, little is also known about the molecular mechanisms underlying the interactions among roots, the root exudate, and other rhizospheric fungi, including the best-studied examples in the genus *Trichoderma* (11). The utilization of plant-derived sugars has been shown to be important for *M. robertsii* to colonize the rhizosphere. A sucrose invertase [MrInV (*Metarhizium robertsii* invertase)] and an oligosaccharide transporter [MRT (*Metarhizium* raffinose transporter)] were shown to utilize sucrose and oligosaccharides derived from roots and are thus important for rhizosphere competency (11, 12). While *M. robertsii* is able to metabolize a wide variety of carbohydrates, some monosaccharides, such as glucose, are preferred by this fungus (13), which are also available in the root exudate (5). Therefore, the utilization of monosaccharides is assumed to be important for rhizosphere competency, which, however, remains to be investigated.

In this study, based on our previously conducted transcriptome sequencing (RNA-Seq) analysis, we found that under all lifestyles, *M. robertsii* constitutively and highly expressed a sugar transporter, and it was then biochemically characterized to be a monosaccharide transporter and designated MST1 (Monosaccharide transporter 1). We further found that the utilization of monosaccharides mediated by MST1 is important for the colonization of the rhizosphere and the rhizoplane. The monosaccharide transporter MST1 and the oligosaccharide transporter MRT act additively to facilitate rhizosphere colonization by *M. robertsii*.

## RESULTS

### Identification of the sugar transporter MST1.

In our previous RNA-Seq analysis (13, 14), we noticed that a gene (MAA_02398) was highly expressed during symbiotic growth on Arabidopsis thaliana roots, saprophytic growth in nutrient-rich SDY medium (Sabouraud dextrose broth supplemented with 1% yeast extract), and infection of insects. MAA_02398 is annotated as a monosaccharide transporter with 538 amino acids, which has 12 transmembrane domains as predicted by TMHMM version 2.0 (15). MAA_02398 is designated MST1 as it is a monosaccharide transporter (see below). BLASTP analysis showed that homologs of MST1 were widely found in many plant-associated fungi such as Epichloë festucae, *Trichoderma* spp., Fusarium spp., and *Claviceps* spp. MST1 homologs were also found in *Ophiocordycipitaceae* fungi such as Hirsutella minnesotensis and Ophiocordyceps australis, which have not been documented to closely associate with plants. Among the top 100 hits identified by NCBI BLASTP analysis, no MST1 homologs were found be in the genome of the *M. robertsii* ARSEF2575 strain, so highly similar paralogs were not present in the genome. The amino acid sequence identity between MST1 and the previously characterized raffinose transporter MRT (12) was only 26% (5e^−34^). A phylogenetic analysis using MST1 and its homologs showed that the gene tree was consistent with the species tree (see Fig. S1 in the supplemental material).

10.1128/mSystems.01277-21.1 FIG S1 Phylogenetic analysis of MST1 and its fungal homologs. Shown is an unrooted maximum likelihood (ML) tree constructed with the protein sequences. Numbers at the nodes represent bootstrap values of neighbor joining (left), ML (middle), and Bayesian posterior probabilities (right). A hyphen indicates no support value from that method. The bar corresponds to the estimated number of amino acid substitutions per site. The GenBank accession number for each sequence is shown after the species name. Download FIG S1, TIF file, 0.5 MB. Copyright © 2021 Dai et al. 2021 Dai et al. https://creativecommons.org/licenses/by/4.0/ This content is distributed under the terms of the Creative Commons Attribution 4.0 International license.

**FIG 1:**
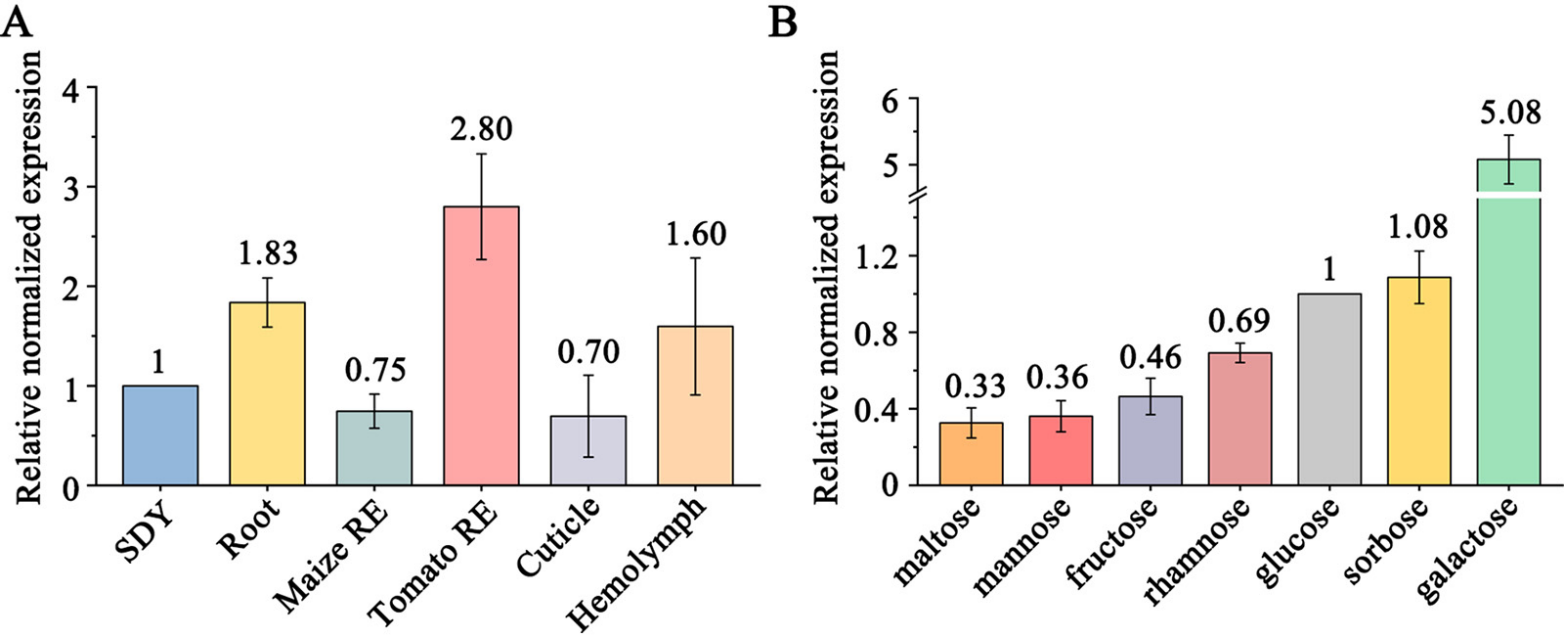
qRT-PCR analysis of *Mst1* expression in the WT *M. robertsii* strain. (A) *Mst1* expression in nutrient-rich SDY medium (SDY), on the roots of *A. thaliana* (Root), in the tomato or maize root exudates (RE), on locust hindwings (Cuticle), and in the G. mellonella hemolymph (Hemolymph). (B) *Mst1* expression in MM containing a sugar (shown at the bottom) as the sole carbon source. qRT-PCR analysis in this study was repeated three times with three replicates, and the values in the panel represent the fold changes of the expression of a gene in treatments compared with the expression levels of the respective controls, which are set to 1. Data are shown as the means ± SE (standard errors).

Quantitative real-time PCR (qRT-PCR) analysis showed that no significant difference in the expression levels of *Mst1* was found for growth in SDY medium, in medium using tomato or maize root exudates as the sole carbon and nitrogen sources, on the roots of Arabidopsis thaliana, on the insect cuticle (locust hindwings), and in the hemolymph (Galleria mellonella larvae) (Fig. 1A). We also assayed the impact of sugars on *Mst1* expression. qRT-PCR analysis showed that the expression level of *Mst1* was higher in medium with galactose than in medium containing glucose, mannose, fructose, rhamnose, sorbose, or maltose (Fig. 1B).

### Biochemical characterization of MST1.

**FIG 2:**
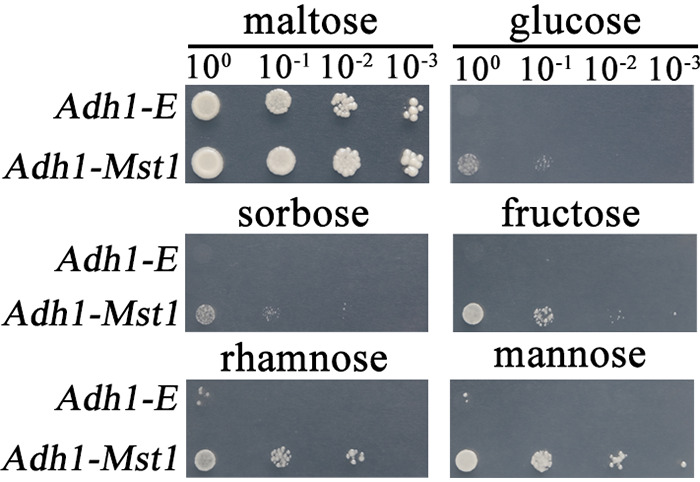
Colony growth of the S. cerevisiae strains on SC medium containing a sugar (shown at the top of each panel) as the sole carbon source. The yeast cell suspension (OD_600_ = 0.5) was serially 10-fold diluted in three steps. *Adh1-Mst1*, a strain with *Mst1* constitutively expressed in the mutant strain EBY.VW4000; *Adh1-E*, a strain constructed with EBY.VW4000 transformed with the empty plasmid pGAD-E. Pictures were taken at 4 days postinoculation.

We then assayed the transport activity and substrate specificity of MST1. To this end, the *Adh1-Mst1* yeast strain was constructed, in which the *Mst1* gene was driven by the promoter of the Saccharomyces cerevisiae *Adh1* gene encoding an alcohol dehydrogenase in the hexose transport-deficient S. cerevisiae mutant EBY.VW4000. In the yeast mutant EBY.VW4000, all 20 endogenous monosaccharide transporter genes are deleted so that it cannot grow on media with hexoses as the sole carbon sources (16). As a control, the *Adh1-E* yeast strain was also constructed by transforming the mutant strain EBY.VW4000 with the empty plasmid pGAD-E. On medium with the disaccharide maltose, the *Adh1-Mst1* and *Adh1-E* yeast strains had the same growth. On medium containing fructose, glucose, or sorbose, the *Adh1-E* strain showed no growth, while the *Adh1-Mst1* strain was able to grow (Fig. 2). The growth of the *Adh1-Mst1* strain was greater than that of the *Adh1-E* strain in medium containing mannose or rhamnose (Fig. 2). No difference in growth was found between the *Adh1-Mst1* and *Adh1-E* strains in medium containing xylose, ribose, sorbitol, mannitol, lyxose, or tagatose (Fig. S2).

10.1128/mSystems.01277-21.2 FIG S2 Colony growth of the *Adh1-Mst1* and *Adh1-E* yeast strains on SC medium containing a sugar (shown at the top of each panel) as the sole carbon source. *Adh1-Mst1* is a strain with *Mst1* constitutively expressed in the mutant strain EBY.VW4000. *Adh1-E* is the negative-control strain constructed with EBY.VW4000 transformed with the empty plasmid pGAD-E. The yeast cell suspension (OD_600_ = 0.5) was serially 10-fold diluted in three steps. Pictures were taken at 4 days postinoculation. Download FIG S2, TIF file, 1.3 MB. Copyright © 2021 Dai et al. 2021 Dai et al. https://creativecommons.org/licenses/by/4.0/ This content is distributed under the terms of the Creative Commons Attribution 4.0 International license.

**FIG 3:**
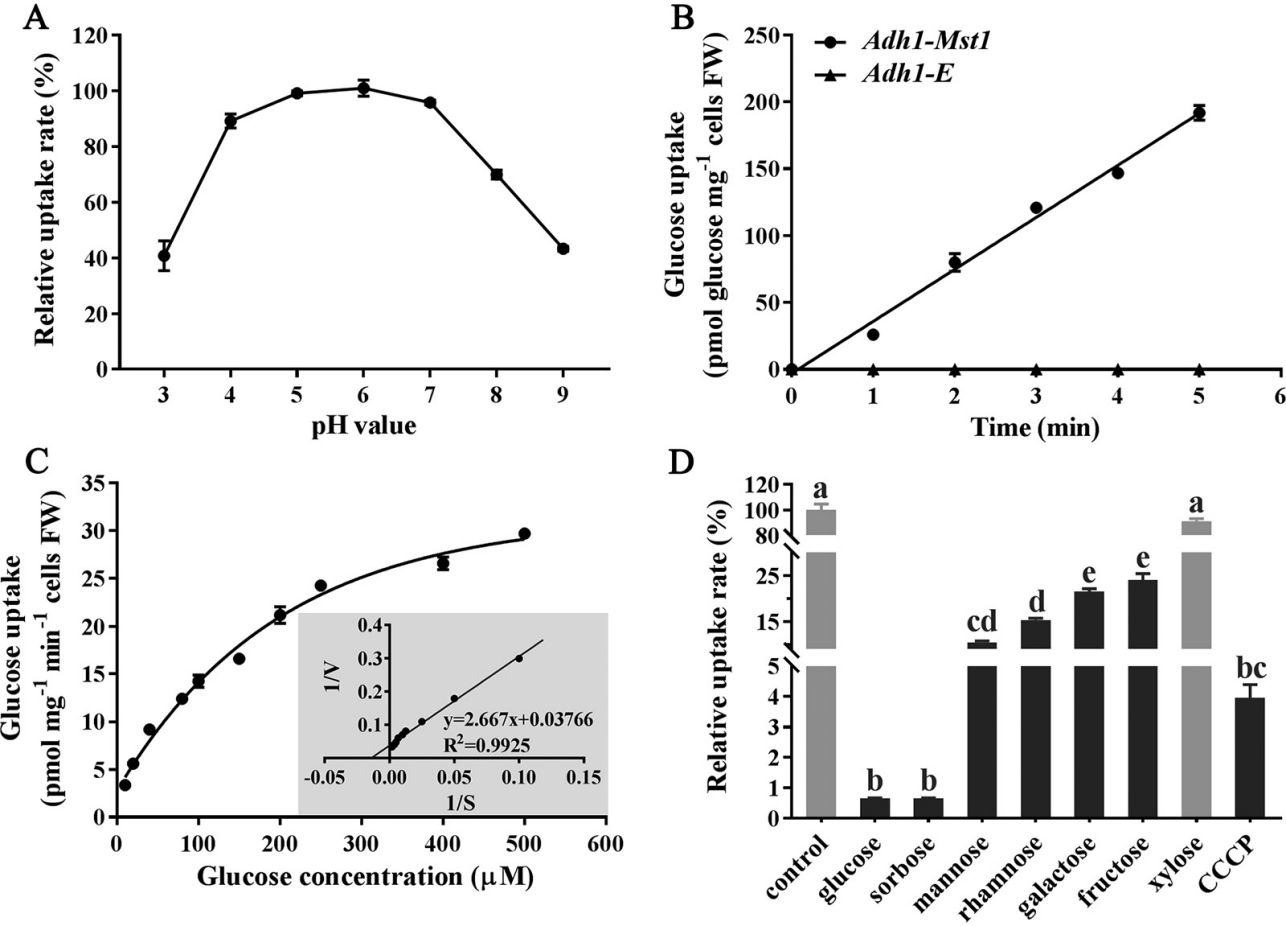
[^14^C]glucose uptake assays of MST1. (A) Relative uptake rate of [^14^C]glucose by the *Adh1-Mst1* yeast strain at different pH values. (B) [^14^C]glucose uptake assays of the *Adh1-Mst1* and *Adh1-E* yeast strains. The pH value was 6. (C) Concentration-dependent [^14^C]glucose uptake and [^14^C]glucose uptake kinetics of MST1. A Lineweaver-Burk plot of a typical *K_m_* determination is presented in the inset. The estimated *K_m_* was 158.3 ± 14.26 μM. The *V*_max_ was 38.03 ± 1.417 pmol mg^−1^ min^−1^. The *K_m_* and *V*_max_ were determined by nonlinear regression using GraphPad Prism 7. (D) Substrate competition assays. A competing sugar (10 mM) was added in a 100-fold molar excess. The *Adh1-Mst1* and *Adh1-E* yeast strains are described above. FW, fresh weight; CCCP, carbonyl cyanide *m*-chlorophenylhydrazone (a transporter inhibitor). The experiments were repeated three times with three replicates per repeat. Data are expressed as the means ± SE. Values with different letters are significantly different (*n* = 3) (*P < *0.05 by Tukey’s test with one-way analysis of variance [ANOVA]).

The transport capacity of MST1 was further analyzed using [^14^C]glucose uptake assays. The optimum pH for MST1 to transport glucose was 6 (Fig. 3A). While the *Adh1-E* control strain was unable to absorb glucose at pH 6.0 (Fig. 3B), the *Adh1-Mst1* strain took up [^14^C]glucose with a *K_m_* value of 158.3 ± 14.26 μM and a maximum uptake rate (*V*_max_) of 38.03 ± 1.417 pmol mg^−1^ min^−1^ (Fig. 3C). Substrate competition assays showed that the uptake of [^14^C]glucose was significantly suppressed by nonradioactive glucose, sorbose, mannose, rhamnose, galactose, and fructose, with glucose and sorbose having the greatest inhibition effects (Fig. 3D). Xylose had no impact on the uptake of [^14^C]glucose. The application of carbonyl cyanide *m*-chlorophenylhydrazone (CCCP), which dissipates the H^+^ gradient and uncouples electron transport from ATP synthesis, significantly inhibited glucose uptake (Fig. 3D).

### Utilization of sugars by MST1 is important for the growth of *M. robertsii*.

We then assayed whether MST1 is involved in *M. robertsii* growth when a monosaccharide was used as the sole carbon source. To this end, we first constructed the Δ*Mst1* deletion mutant and its complemented strain *C-*Δ*Mst1* (Fig. S3).

10.1128/mSystems.01277-21.3 FIG S3 Construction of gene deletion mutants. (A) Schematic diagram of gene deletion based on homologous recombination. (B) Confirmation of the deletion mutant of *Mst1* by PCR. D, deletion mutant; WT, wild-type strain. (Top) PCR was conducted with the primers Bar-up and CF-2; (bottom) PCR was conducted with the primers CF-1 and CF-2. The relative positions of primers are shown in panel A. (C) Confirmation of the complementation of the Δ*Mst1* strain. C, complemented strain. PCR was conducted with primers ORF-5 and ORF-3. (D) Confirmation of the construction of the Δ*Mst1*::Δ*Mrt* double-gene-deletion mutant by deleting *Mst1* in the Δ*Mrt* mutant. Download FIG S3, TIF file, 0.4 MB. Copyright © 2021 Dai et al. 2021 Dai et al. https://creativecommons.org/licenses/by/4.0/ This content is distributed under the terms of the Creative Commons Attribution 4.0 International license.

**FIG 4:**
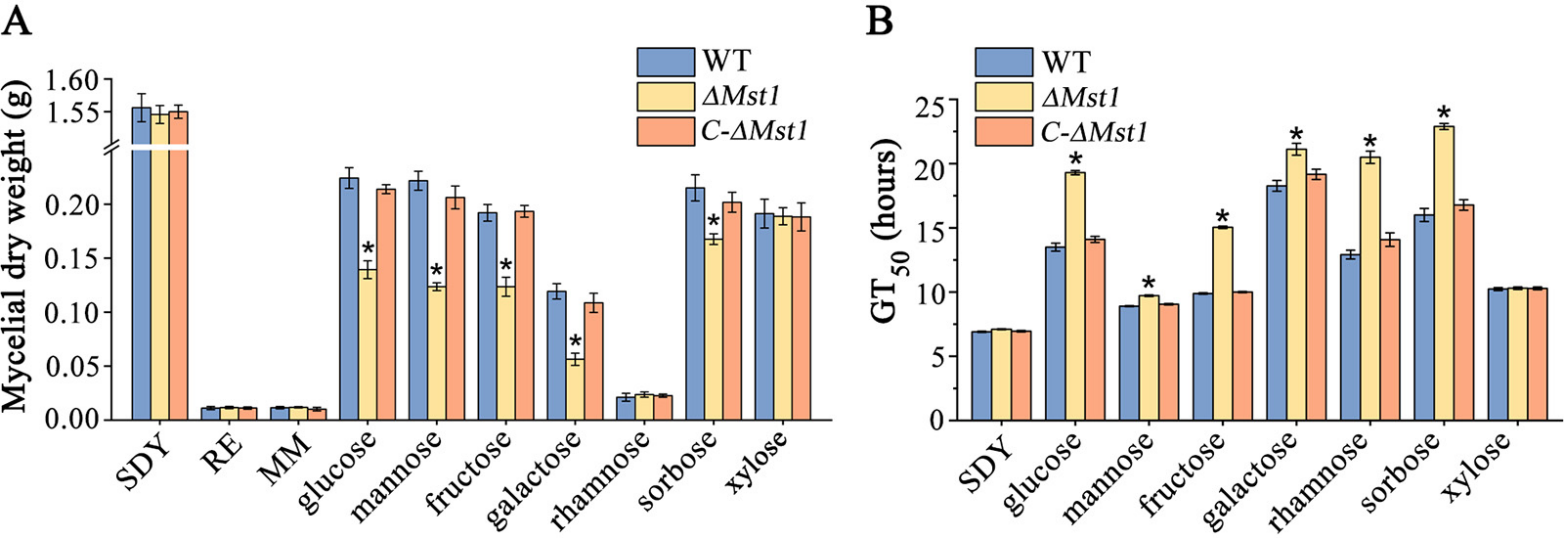
MST1 is involved in the mycelial growth and germination of *M. robertsii*. (A) Dry weight of mycelium grown in different media. (B) GT_50_ (time taken for 50% of spores to germinate) values in different media. MM, M100 medium without a carbon source; SDY, nutrient-rich SDY medium; RE, medium with the tomato root exudate as the sole carbon and nitrogen source; WT, wild-type strain; Δ*Mst1*, deletion mutant of *Mst1*; *C*-Δ*Mst1*, complemented strain of the Δ*Mst1* mutant. Other media included MM supplemented with a sugar (shown at the bottom) as the sole carbon source. The experiments were repeated three times with three replicates per repeat. Data are expressed as the means ± SE. For each medium, values with an asterisk are significantly different from the others (*n* = 3) (*P < *0.05 by Tukey’s test with one-way ANOVA).

In nutrient-rich SDY medium, no significant difference in mycelial dry weight was found among the wild-type (WT) strain, the Δ*Mst1* deletion mutant, and its complemented strain *C-*Δ*Mst1* (Fig. 4A). In medium with glucose, mannose, fructose, galactose, or sorbose, the dry weight of the WT mycelia was significantly higher than that of the Δ*Mst1* mutant, but no difference was found between the WT and *C-*Δ*Mst1* strains (Fig. 4A). Although MST1 can transport rhamnose, *M*. *robertsii* grew very slowly in medium containing this sugar, and no difference in mycelial growth was found among the WT, Δ*Mst1* mutant, and *C-*Δ*Mst1* strains (Fig. 4A). Likewise, mycelial growth was also slow in the medium using the tomato root exudate (0.01 mg/ml) as the sole carbon and nitrogen source, and no significant difference in dry mycelial weight was found among the three strains. In the medium containing xylose that MST1 could not transport (Fig. 3D), no significant difference in mycelium growth was found among the WT, Δ*Mst1* mutant, and *C-*Δ*Mst1* strains (Fig. 4A).

The deletion of *Mst1* significantly slowed down the spore germination of *M. robertsii* in the medium containing mannose, glucose, fructose, galactose, sorbose, or rhamnose, and no difference in the GT_50_ (time taken for 50% of spores to germinate) was found between the WT strain and the *C-*Δ*Mst1* complemented strain (Fig. 4B). In the medium containing xylose that MST1 could not transport, no significant difference in germination was found among the WT, Δ*Mst1* mutant, and *C-*Δ*Mst1* strains. No significant difference in germination was found among these three strains in the nutrient-rich SDY medium (Fig. 4B). In the medium with the tomato root exudate (0.01 mg/ml) as the sole carbon and nitrogen source, 35.6% ± 1.31% of the WT spores and 18.7% ± 0.97% of the Δ*Mst1* spores germinated at 36 h postinoculation, and no significant difference was found between the WT strain and the *C-*Δ*Mst1* complemented strain (33.7% ± 1.01%).

Bioassays showed that there was no significant difference in pathogenicity to G. mellonella insects among the WT, Δ*Mst1*, and *C-*Δ*Mst1* strains (Fig. S4).

10.1128/mSystems.01277-21.4 FIG S4 Survival curves of the G. mellonella larvae inoculated with the WT, Δ*Mst1*, and *C-*Δ*Mst1* strains. Data are expressed as the means ± SE. The bioassay experiment was repeated three times. Download FIG S4, TIF file, 0.3 MB. Copyright © 2021 Dai et al. 2021 Dai et al. https://creativecommons.org/licenses/by/4.0/ This content is distributed under the terms of the Creative Commons Attribution 4.0 International license.

### MST1 and MRT act collectively to colonize the rhizosphere and rhizoplane.

**FIG 5:**
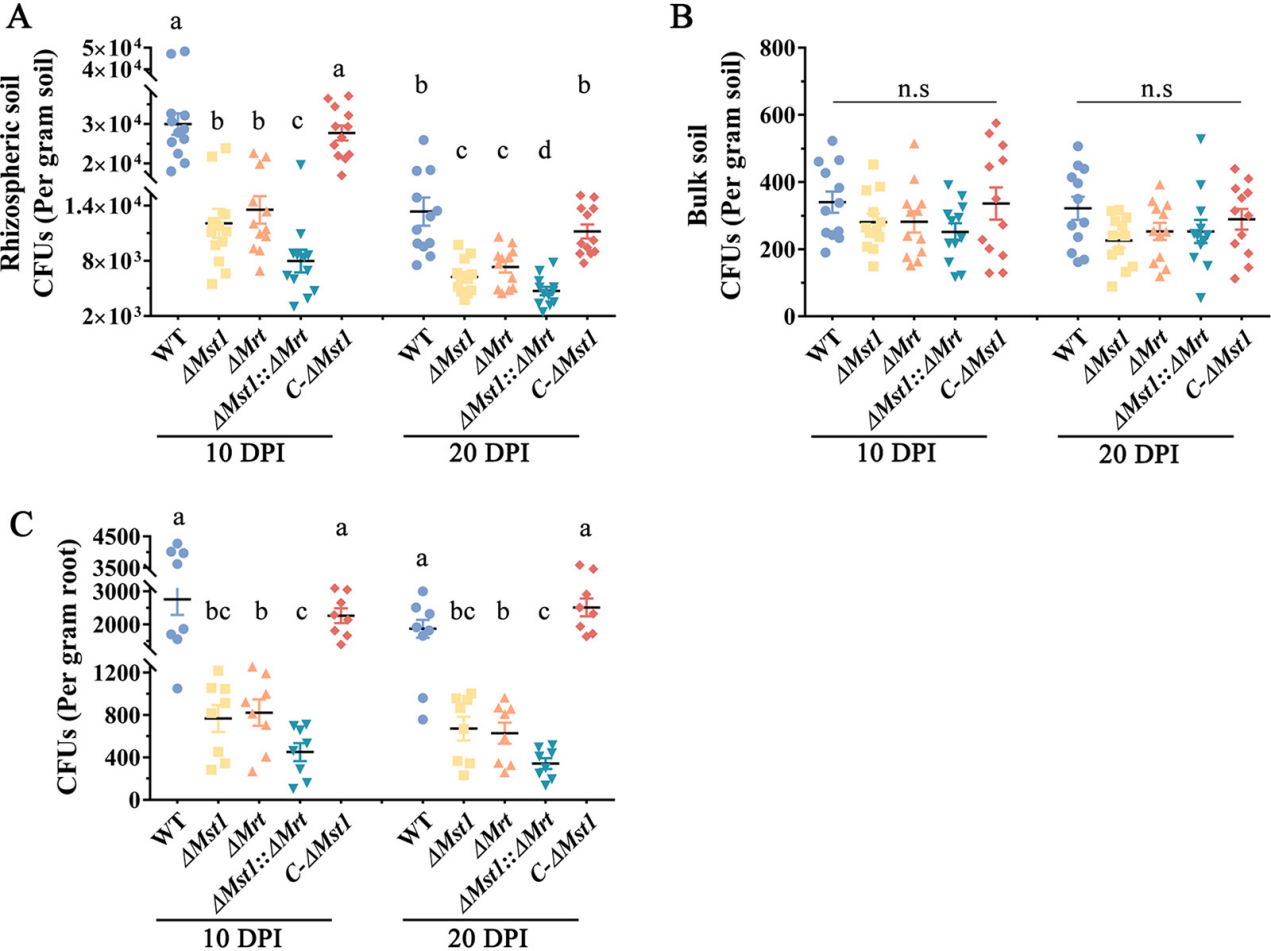
Colonization of the maize rhizosphere or rhizoplane by *M. robertsii* at 10 and 20 days postinoculation (DPI). CFU per gram of rhizospheric soil (A), bulk soil (B), and roots (rhizoplane) (C) are shown. These assays were repeated six times with two replicates per repeat. Values with different letters are significantly different (*P < *0.05 by a Mann-Whitney test). n.s, no significant difference. Data are expressed as the means ± SE. WT, wild-type strain; Δ*Mst1*, deletion mutant of *Mst1*; *C*-Δ*Mst1*, complemented strain of the Δ*Mst1* mutant; Δ*Mrt*, deletion mutant of *Mrt*; Δ*Mst1*::Δ*Mrt*, double-gene-deletion mutant of *Mst1* and *Mrt*.

We then assayed whether the monosaccharide transporter MST1 is involved in the colonization of the rhizosphere and rhizoplane. The oligosaccharide transporter MRT was shown to contribute to the colonization of the rhizosphere (12), so the Δ*Mst1*::Δ*Mrt* double-gene-deletion mutant was constructed to investigate whether MST1 and MRT contribute additively to rhizosphere colonization (Fig. S3). The ability to colonize the maize rhizosphere was analyzed by counting CFU in rhizospheric soil samples. At 10 and 20 days postinoculation (dpi), counts of the WT strain were significantly higher than those of the Δ*Mst1* and Δ*Mrt* single-gene-deletion mutants, which in turn were higher than those of the Δ*Mst1*::Δ*Mrt* double-gene-deletion mutant (Fig. 5A). Counts of the WT strain were 3.7-fold and 2.6-fold higher than those of the Δ*Mst1*::Δ*Mrt* mutant at 10 and 20 dpi, respectively (Fig. 5A). No difference in rhizosphere colonization was found between the WT strain and the *C*-Δ*Mst1* complemented strain (Fig. 5A). In the bulk soil, no significant differences in the numbers of CFU were found among all tested strains (Fig. 5B).

For the endophytic growth assay, we could not obtain any Metarhizium colonies, so *M. robertsii* appeared not to grow endophytically in the maize roots. For rhizoplane colonization, at 10 and 20 dpi, the number of CFU of the WT strain was significantly higher than those of the Δ*Mst1* and Δ*Mrt* single-gene-deletion mutants (Fig. 5C). Although the mean number of CFU of the Δ*Mst1*::Δ*Mrt* double-deletion mutant was lower than those of the single-gene-deletion mutants, a significant difference was found only between the Δ*Mst1*::Δ*Mrt* and Δ*Mrt* strains (Fig. 5C). The numbers of WT CFU were 5.2-fold and 5.3-fold higher than those of the Δ*Mst1*::Δ*Mrt* mutant at 10 and 20 dpi, respectively (Fig. 5C). No significant difference in rhizoplane colonization was found between the WT strain and the *C-*Δ*Mst1* complemented strain.

We also investigated the effects of the symbiotic interaction between maize and *M. robertsii* on plant growth, which was shown by four traits: plant fresh and dry weights (root, above-ground part, and whole plant), plant height, length of principal roots, and number of lateral roots. At 20 dpi, no significant differences in all four assayed traits were found between the plants inoculated with the WT strain and those (negative control) that were not inoculated with a fungal strain; no significant differences were also found among the plants treated with the WT strain, the mutants (Δ*Mrt*, Δ*Mst1*, and Δ*Mst1*::Δ*Mrt*), and the C-Δ*Mst1* complemented strain (Fig. S5).

10.1128/mSystems.01277-21.5 FIG S5 Impact of the interaction between *M. robertsii* and maize on plant growth. (A) Length of principal roots. (B) Number of lateral roots. (C) Fresh weight and dry weight of the above-ground part of a plant, its roots, and the whole plant. In panels A to C, data were collected 20 days after inoculation of spores into the plant roots. (D) Plant height was measured daily from day 1 after inoculation of spores into the plant roots. These assays were repeated six times with two replicates per repeat. n.s, no significant difference. Data are expressed as the means ± SE. WT, wild-type strain; Δ*Mst1*, deletion mutant of *Mst1*; *C*-Δ*Mst1*, complemented strain of the Δ*Mst1* mutant; Δ*Mrt*, deletion mutant of *Mrt*; Δ*Mst1*::Δ*Mrt*, double-gene-deletion mutant of *Mst1* and *Mrt*; CK, control plants that were not treated with fungi. Download FIG S5, TIF file, 1.8 MB. Copyright © 2021 Dai et al. 2021 Dai et al. https://creativecommons.org/licenses/by/4.0/ This content is distributed under the terms of the Creative Commons Attribution 4.0 International license.

## DISCUSSION

The fungal population in the rhizosphere is supported by a very complex mixture of relatively labile organic compounds such as sugars, amino acids, and phenolics in the root exudate. Genomic analysis has shown that rhizospheric fungi usually have a large number of genes that are potentially involved in the utilization of root-derived carbon sources. For example, 29 sugar transporters were found in the genome of the fungus *M. robertsii* (17). However, only the oligosaccharide transporter MRT has so far been functionally characterized to be important for rhizosphere colonization by *M. robertsii* (12). In fact, to the best of our knowledge, few sugar transporters have been functionally characterized as contributing to the development of a symbiotic relationship between plants and other fungi.

In this study, we showed that a monosaccharide (MST1) is important for *M. robertsii* to colonize the maize rhizosphere and rhizoplane. We further found that MST1 acts additively with the oligosaccharide transporter MRT to colonize the rhizosphere. The major importance of the sugar transporters MST1 and MRT in the colonization of the maize rhizosphere is consistent with the constituents of the maize root exudate, which is comprised of sugars (70%), phenolics (18%), organic acids (7%), and amino acids (3%) (6). The discrepancy between the collective contributions of MRT and MST1 to rhizoplane and rhizosphere colonization could be due to the difference in the available nutrients between these two areas. For other plant species that have different relative amounts of sugars, phenolics, and amino acids, the collective roles of MST1 and MRT in rhizosphere and rhizoplane colonization could be different from those for maize. It has been documented that the WT *M. robertsii* strain had beneficial effects on maize in a farm field by increasing leaf collar formation, stalk length, average ear biomass, and average stalk and foliage biomass; deletion of the *Mrt* gene reduced such beneficial effects (18). In this study, we found that in a growth chamber, the WT *M. robertsii* strain had no obvious beneficial effects on maize plants, and no difference was found between the WT strain and the mutants (Δ*Mrt*, Δ*Mst1*, and Δ*Mst1*::Δ*Mrt*). At least two differences in the experimental design could explain the discrepancy between the results of this study and those of the previous one (18). One is that the growth conditions were different between these two studies; in this study, maize was grown in a growth chamber in sterilized soil, while the plants were grown in a farm field in the previous study. The other one is that the times for recording the effects of *M. robertsii* on plant growth were different. Due to the space limitation with the growth chamber used in this study, plant growth was recorded for only 20 days, while in the previous study, plant growth was monitored for a much longer time, with the beneficial effects of *M. robertsii* on plant growth being observed in the late stage. Therefore, the beneficial effects of *M. robertsii* on maize plants are dependent on many factors such as the growth conditions for the plants and the development stage of the plants.

The *Mst1* gene is highly expressed during all three lifestyles: saprophytic, plant root symbiotic, and insect pathogenic. The *Mst1* deletion mutant was impaired in plant-symbiotic growth and saprophytic growth in medium with a monosaccharide that MST1 can transport. However, the deletion of *Mst1* had no significant impact on pathogenicity against insects. One possible explanation is that the functions of MST1 are compensated for by other genes in the Δ*Mst1* deletion mutant during infection of insects. Homologs of MST1 were found in many plant-associated fungi and those that have not been documented to closely associate with plants, further suggesting that they contribute to the acquisition of sugars not only from plants but also from other resources.

In conclusion, in the plant-symbiotic fungus *M. robertsii*, we identified a novel monosaccharide transporter (MST1) that is important for rhizoplane colonization and acts additively with the previously characterized oligosaccharide transporter MRT to facilitate rhizosphere colonization. Homologs of MST1 were widely found in many other plant-symbiotic fungi, suggesting that the utilization of plant-derived sugars by MST1 homologs could be important for these fungi to interact with their respective plant hosts. Our work significantly advances insights into the mechanisms for the development of a symbiotic relationship between plants and fungi.

## MATERIALS AND METHODS

### Microorganisms and plants.

*M. robertsii* ARSEF2575 was obtained from the Agricultural Research Service Collection of Entomopathogenic Fungi. Escherichia coli strain DH5α and Agrobacterium tumefaciens AGL1were used for plasmid construction and fungal transformation, respectively (19). The S. cerevisiae mutant strain EBY.VW4000 was used for analyzing the transport activity of MST1 (16).

A. thaliana ecotype Columbia (Col-0) seeds were obtained from the ABRC center at Ohio State University (Columbus, OH). Maize (Zea mays) seeds (Zhongnongtian 488) were commercially purchased (Beijing Huanai Agricultural Development Co., Ltd., Beijing, China).

### Gene deletion.

Gene deletion based on homologous recombination was conducted as previously described (19). The master plasmids Ppk2-OSCAR-GFP and pA-Bar were used to construct the *Mst1* deletion plasmid using Gateway Bp Clonase II enzyme mix (Invitrogen, USA). All DNA fragments in this study were cloned using PCR with high-fidelity *Taq* DNA polymerase (KOD plus neo). All PCR products were confirmed by sequencing. All primers used in this study are listed in Table S1 in the supplemental material. The Δ*Mrt*::Δ*Mst1* double-gene-deletion mutant was constructed by deleting *Mst1* in the Δ*Mrt* deletion mutant (12). To this end, another *Mst1* deletion plasmid using the *Sur* gene as a selection marker was constructed. To complement the Δ*Mst1* deletion mutant, a DNA fragment containing its promoter and ORF (open reading frame) and the downstream terminator region was cloned by PCR using *M. robertsii* genomic DNA as the DNA template, which was then recombined into the plasmid pPK2-NTC-GFP (20). The resulting plasmid was then transferred into the Δ*Mst1* mutant to construct the *C-*Δ*Mst1* complemented strain.

10.1128/mSystems.01277-21.6 TABLE S1 Primers used in this study. Download Table S1, DOCX file, 0.02 MB. Copyright © 2021 Dai et al. 2021 Dai et al. https://creativecommons.org/licenses/by/4.0/ This content is distributed under the terms of the Creative Commons Attribution 4.0 International license.

### Assays of MST1 transport activity with S. cerevisiae.

To assay the transport activity of MST1, the *Mst1* expression plasmid pGAD-Mst1 was first constructed. The plasmid pGADT7 (Clontech, Japan) was digested with the restriction enzyme HindIII to remove the portion corresponding to the simian virus 40 (SV40) nuclear localization signal (NLS) and the GAL4 activation domain (AD) and then self-ligated to produce the empty plasmid pGAD-E. The coding sequence of MST1 was cloned by PCR, and the resulting PCR product was digested with HindIII and cloned into the plasmid pGAD-E to produce the plasmid pGAD-Mst1, in which the *Mst1* gene was driven by the constitutive promoter of the S. cerevisiae *Adh1* gene. The plasmids pGAD-Mst1 and pGAD-E were then transformed into the yeast strain EBY.VW4000 using the Yeastmaker yeast transformation system kit (TaKaRa, Japan) to produce the *Adh1-Mst1* and *Adh1-E* strains, respectively. Transformants were selected on synthetic complete (SC) medium (0.67% yeast nitrogen base, 0.069% Leu dropout mix, 2% agar) supplemented with maltose (2%).

To assay the utilization of monosaccharides by a recombinant EBY.VW4000 yeast strain, a single colony was inoculated into liquid SC medium with maltose and incubated at 30°C for 16 h with shaking at 220 rpm. The cells were then harvested by centrifugation at 4,000 rpm for 5 min, washed twice using sterile water, and resuspended in an NaCl solution (0.9%) to achieve an optical density at 600 nm (OD_600_) of 0.5. The cell suspension was subjected to 10-fold serial dilutions, and an aliquot (3 μl) was spotted onto the SC medium plate supplemented with a monosaccharide (2%) as the sole carbon source. After incubation at 30°C for 4 days, the growth of the yeast cells was recorded.

Glucose uptake by a recombinant yeast strain was assayed as previously described (21). Briefly, yeast cells were grown in liquid SC medium to achieve an OD_600_ value of ∼1.0, i.e., logarithmic phase, and 10 ml of the culture was centrifuged at 4,000 rpm for 5 min to collect cells, which were then washed with ice-cold water. The cell pellet was then weighed and resuspended in 1 ml of cold transport buffer (50 mM phosphate buffer, pH values ranging from 3 to 9). The cell suspension was then aliquoted (100 μl) into a 1.5-ml tube, which was placed on ice for later use. For glucose uptake assays, 100 μl of the yeast cell suspension and an equal volume of a glucose solution {98 μl of unlabeled glucose solution (10, 20, 40, 80, 100, 150, 200, 250, 400, or 500 μM) and 2 μl of 2-[^14^C(U)]d-glucose solution (274 mCi/mmol)} were mixed and incubated at 30°C at 200 rpm for the allotted time. 2-[^14^C(U)]d-glucose was commercially purchased (catalog number NEC720A; Perkin-Elmer, USA). After incubation, the cells were immediately collected on a glass cellulose membrane (pore size of 0.22 μm) by filtration under a vacuum and then washed with ample ice-cold water. The cellulose membrane with the yeast cells was then incubated in a scintillation cocktail overnight to lyse the cells, and the radioactivity intensity was measured by using the Tri-CARB 4910TR liquid scintillation counter (Perkin-Elmer, USA). The amount of glucose transported into the yeast cells is shown as picomoles of glucose per milligram of cells.

For substrate competition assays, a competing nonradioactive sugar was supplied in a 100-fold molar excess to the glucose uptake mixture as described above.

To assay the impact of the pH gradient on the transport activity of MST1, carbonyl cyanide *m*-chlorophenylhydrazone (CCCP) (50 μM) was added to the glucose uptake mixture as described above. All experiments were repeated three times.

The sugars used in this study are d-glucose, d-mannose, d-fructose, d-galactose, d-xylose, d-lyxose, d-tagatose, d-mannitol, d-sorbitol, l-rhamnose, l-sorbose, and l-arabinose.

### Spore germination assay.

The germination rate is shown as the GT_50_. To do this, 60 μl of a spore suspension (4 × 10^7^ spores ml^−1^) was inoculated into nutrient-rich SDY medium (3 ml) or minimal medium (MM) (M100 medium with glucose excluded) supplemented with different sugars (1%). M100 medium was described in our previous work (19). After 4 h of incubation at 26°C, the number of germinated spores was counted every 2 h.

To assay the utilization of the root exudate by *M. robertsii*, spores were inoculated into MM supplemented with the tomato root exudate (0.01 mg ml^−1^) (22). After 36 h of incubation at 26°C, the number of germinated spores was determined.

All experiments were repeated three times with three biological replicates.

### Mycelial growth assay.

To assay mycelial growth in MM supplemented with different sugars, 1 × 10^8^ spores were inoculated into SDY medium (100 ml). After 36 h of shaking at 220 rpm at 26°C, the mycelium was collected by filtration and washed three times with sterile water, which (0.2 g [wet weight]) was inoculated into 50 ml of MM plus a monosaccharide (1%). After incubation at 26°C for 2 days with gentle shaking, the mycelium was harvested by filtration and lyophilized on a freeze-drier (Labcott, USA) for dry weight determination.

### qRT-PCR analysis.

Total RNA in the mycelium was extracted with the TRIzol reagent (Life Technologies, USA). cDNAs were synthesized with total RNAs using the ReverTra Ace qPCR (quantitative PCR) RT master mix (Toyobo, Osaka, Japan). qPCR analysis was conducted using the Thunderbird SYBR qPCR mix without ROX (Toyobo). *gpd* and *act* were used as the reference genes (23). The relative expression level of a gene was calculated using the comparative threshold cycle (2^−ΔΔ^*^CT^*) method (13). All qRT-PCR experiments were repeated three times. The primers for qRT-PCR analysis are shown in Table S1.

To prepare the mycelium grown in SDY medium, conidia (10^8^) were inoculated into 100 ml of the medium and incubated at 26°C for 36 h with shaking at 220 rpm, and the mycelium was then collected by filtration for RNA extraction. To analyze *Mst1* expression in MM with a sugar as the sole carbon source, the mycelium grown in SDY medium as described above was collected, washed with ample sterile water three times, and then (0.2 g [wet weight]) incubated at 26°C for 2 h in 100 ml of MM with a sugar. To prepare the mycelium grown in root exudates, 0.2 g of the mycelium grown in SDY medium was incubated at 26°C for 2 h in water supplemented with tomato or maize root exudates (0.01 mg/ml). The tomato root exudate in Hoagland’s nutrient solution was prepared as previously described (22), and the maize root exudate was made as previously described (24). The mycelia grown in the hemolymph of G. mellonella larvae and on locust hindwings were prepared as previously described (13, 14).

### Assays of colonization of the rhizosphere and rhizoplane.

Briefly, maize seeds were surface sterilized with a sodium hypochlorite (NaOCl) solution (0.4%) for 5 min and rinsed with sterile distilled water. The seeds were further sterilized in a hydrogen peroxide solution (15%) for 10 min, followed by three washes with sterile distilled water. The sterilized seeds were kept on water agar (2%) at 4°C overnight to synchronize germination and then placed at 24°C until germination. A germinated seedling was then planted in sterile soil in a porous fabric pocket (14 cm in height by 9 cm in diameter) in a plastic garden pot (14 cm in height by 15 cm in diameter). The fabric pocket prevented the roots from occupying the room outside the pocket. The sterile soil contained peat soil (catalog number 876; Klasmann-Deilmann) and domestic nutrient soil mixed with vermiculite. Ten milliliters of the conidial suspension (9 × 10^5^ conidia ml^−1^) was inoculated into the soil in the fabric pocket, and the plant was then cultivated in a growth chamber at 25°C with a photoperiod of 16 h of light/8 h of darkness.

At 10 days and 20 days postinoculation, colonization of the rhizosphere and rhizoplane and endophytic growth were assayed. To assay rhizoplane colonization and endophytic growth simultaneously, the roots were washed with sterile water to completely remove rhizospheric soil attached to the roots and then dried with sterile tissue paper, weighed, and homogenized in 1 ml of a Triton X-100 solution (0.05%). The resulting homogenate (100 μl) was then plated onto Metarhizium selective medium (12) to allow fungal growth. The number of CFU was counted after 10 days of incubation, and the ability for rhizoplane colonization and endophytic growth is shown as the number of CFU. To assay endophytic growth only, the roots were further sterilized with an NaOCl solution for 10 s to remove fungi in the rhizoplane, rinsed with ample sterile water three times, homogenized, and plated onto Metarhizium selective medium to allow fungal growth. As described above (Fig. 5), no endophytic growth was found in this study.

Colonization of the rhizosphere was assayed as previously described (12, 25), which is shown by the number of CFU in rhizospheric soil samples. The soil adjacent to the pot wall, which was around 5 cm away from the porous fabric pocket, was collected as the bulk soil.

The experiment was repeated six times with two replicates per repeat.

### Insect bioassays.

Bioassays were conducted using G. mellonella larvae as described previously (26). Insects were immersed in a spore suspension (3 × 10^7^ conidia ml^−1^) with gentle shaking for 15 s, individually placed into a small plastic cup (diameter of 5 cm), and incubated at 26°C with ∼90% humidity. Insect mortality was recorded daily. All bioassays were repeated three times with 40 insects per repeat.

### Statistical analysis.

Tukey’s honestly significant difference test in the OriginPro 8.5 program was used in this study (OriginLab, USA).

### Data availability.

The data that support the findings of this study are available from the corresponding author upon reasonable request.
