# Supplementary figures and images for: The Sugar Transporter MST1 Is Involved in Colonization of Rhizosphere and Rhizoplane by Metarhizium robertsii
Source: mSystems. 2021 Dec 14;6(6):e01277-21. doi: 10.1128/mSystems.01277-21 (PMC8670370; doi:10.1128/mSystems.01277-21)

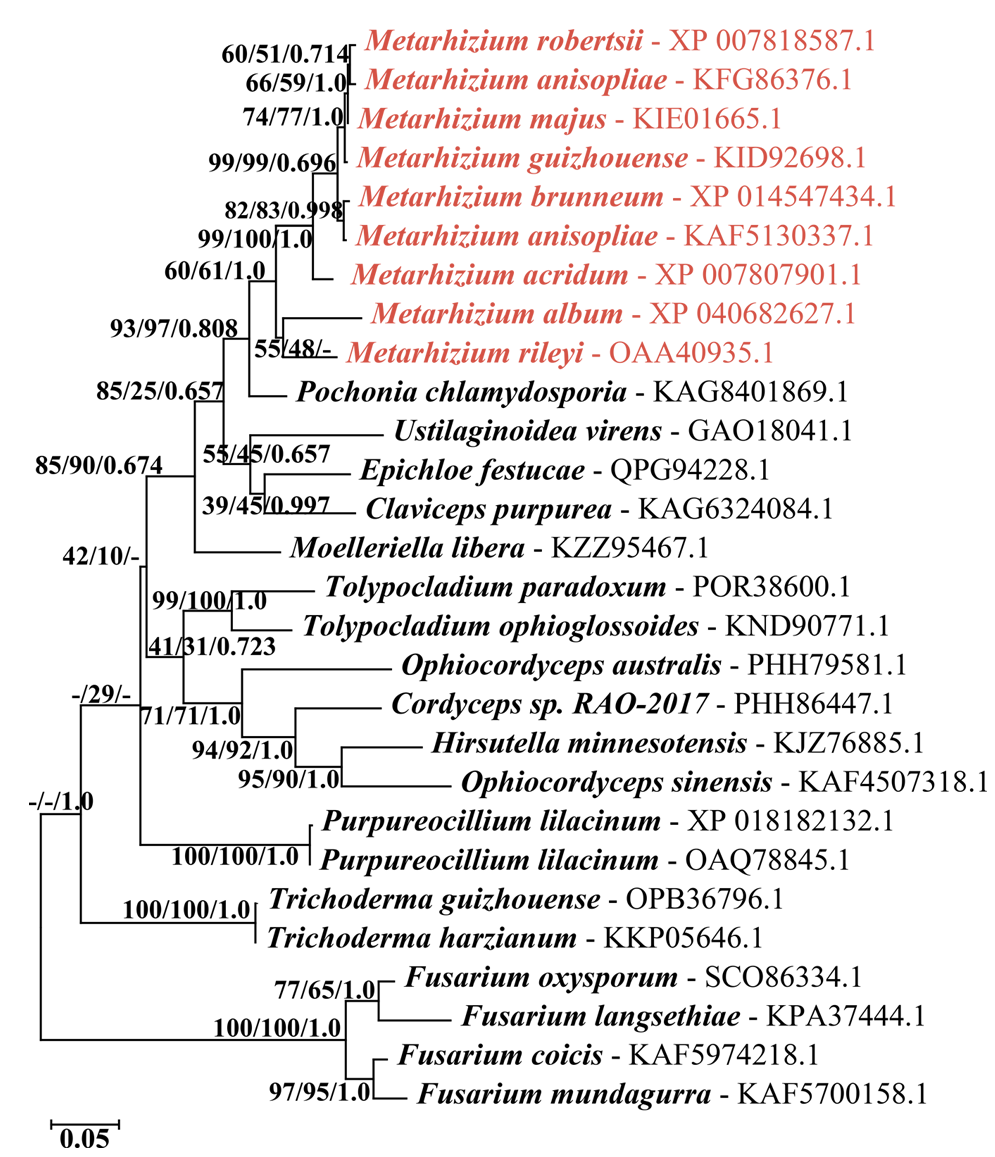

Supplement: FIG S1 [file msystems.01277-21-sf001.tif]

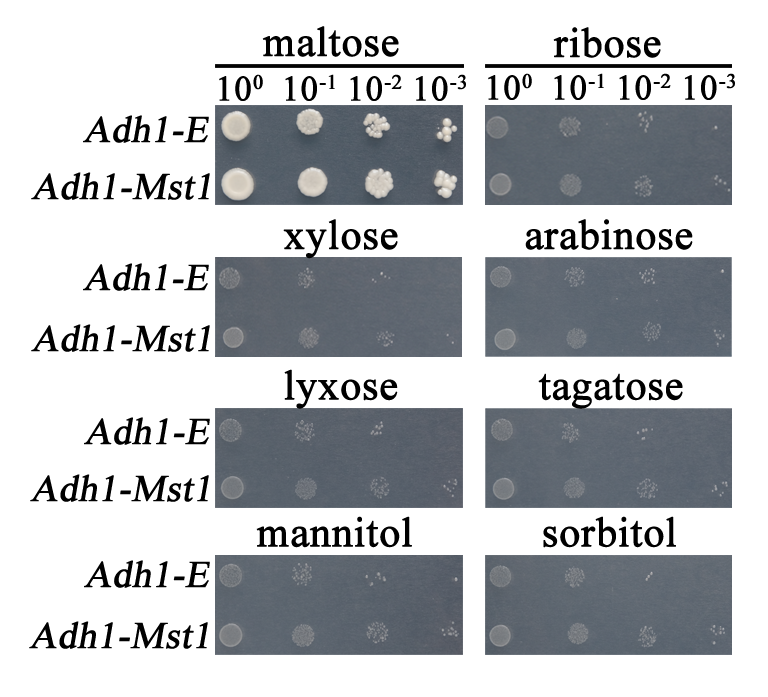

Supplement: FIG S2 [file msystems.01277-21-sf002.tif]

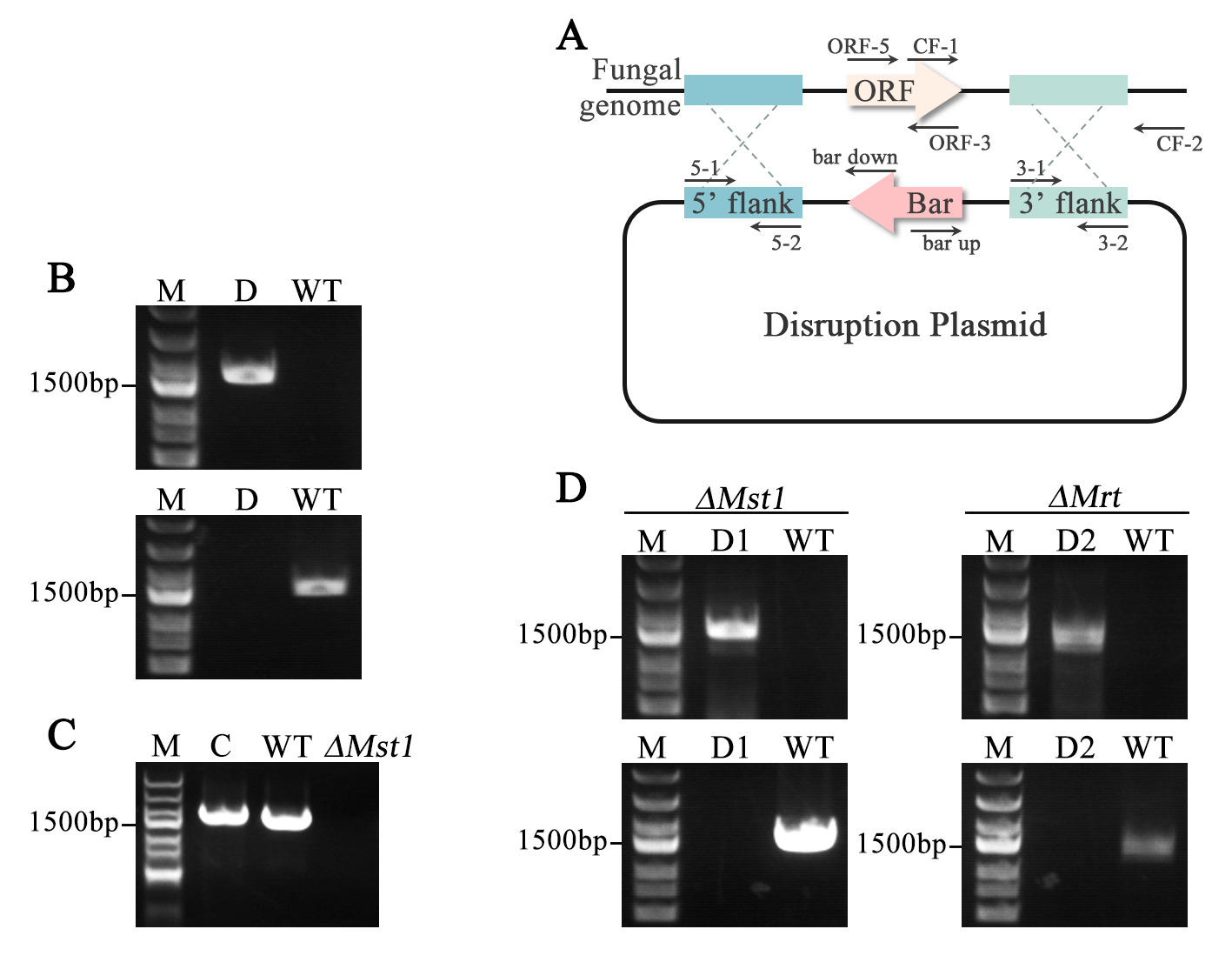

Supplement: FIG S3 [file msystems.01277-21-sf003.tif]

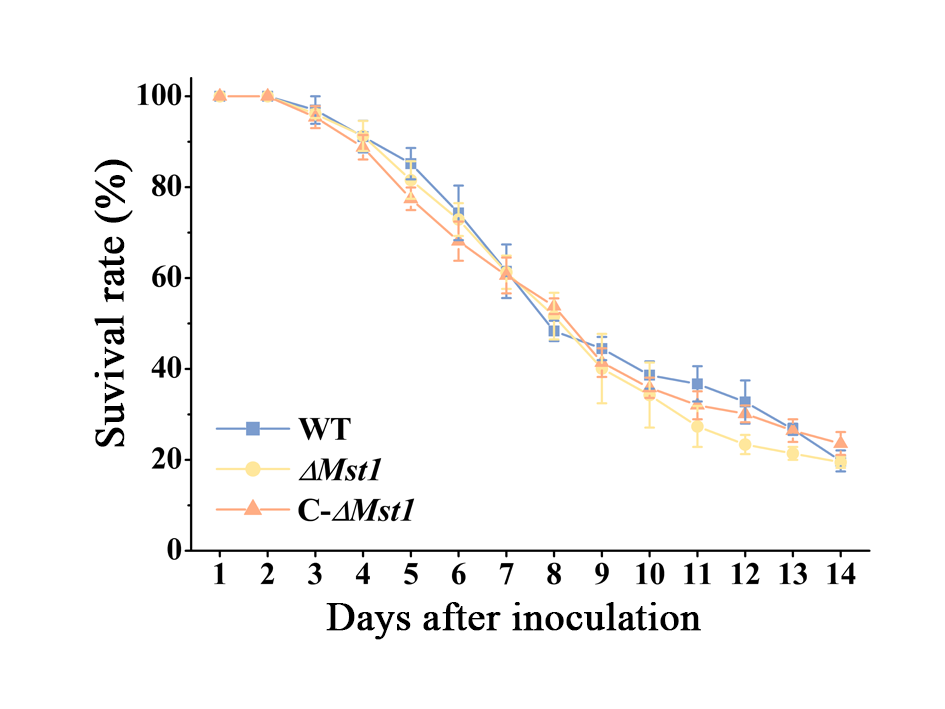

Supplement: FIG S4 [file msystems.01277-21-sf004.tif]

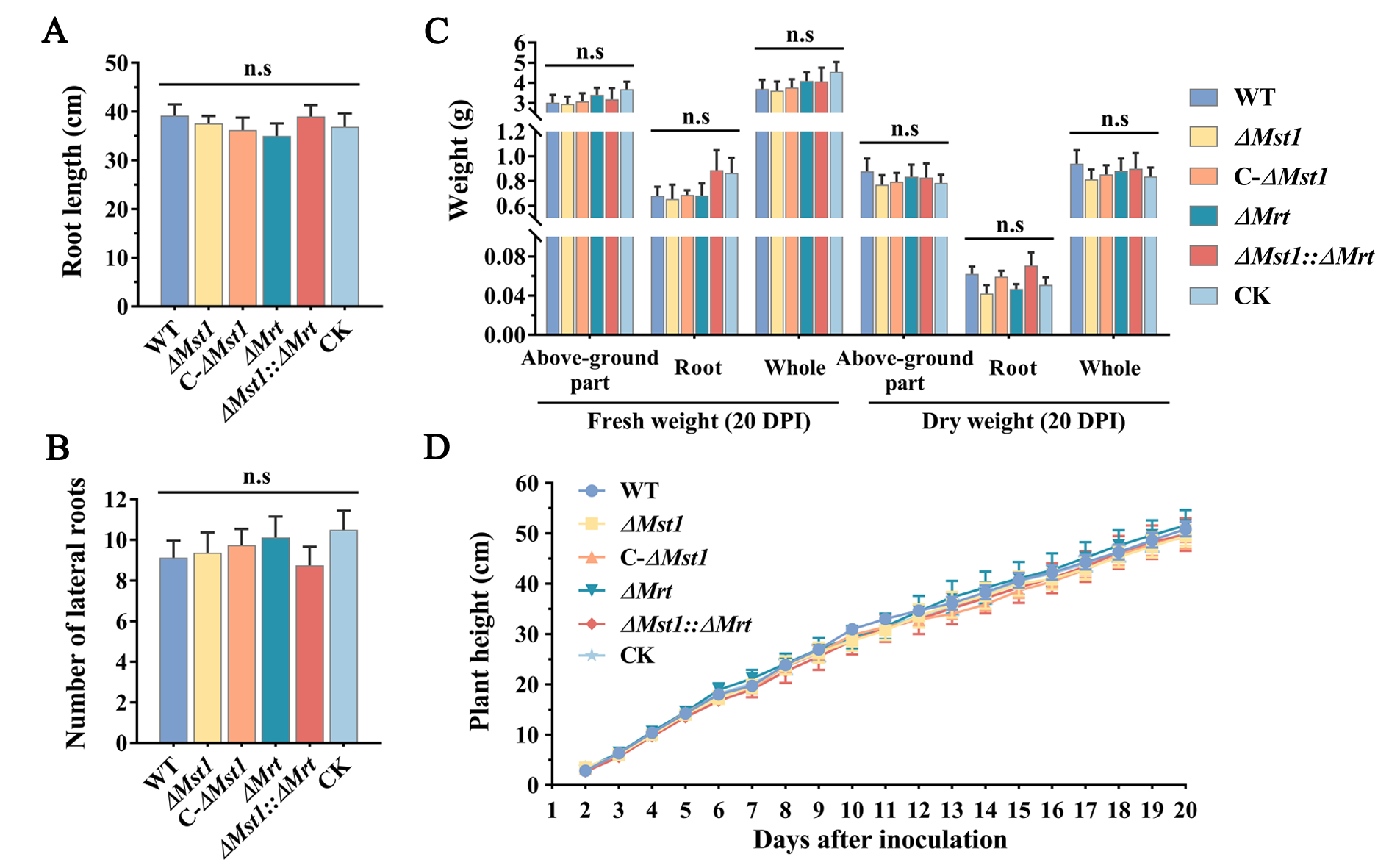

Supplement: FIG S5 [file msystems.01277-21-sf005.tif]
