# Supplementary material for: The Sugar Transporter MST1 Is Involved in Colonization of Rhizosphere and Rhizoplane by Metarhizium robertsii
Source: mSystems. 2021 Dec 14;6(6):e01277-21. doi: 10.1128/mSystems.01277-21 (PMC8670370; doi:10.1128/mSystems.01277-21)
Supplement: TABLE S1 [file msystems.01277-21-st001.docx]

**Table S1. Primers used in this study**

| **Primer** | | | **Sequence** | **Usage** |
| --- | --- | --- | --- | --- |
| DMst1-5-1 | | | GGGGACAGCTTTCTTGTACAAAGTGGAATGGTAGAGATGTGCAAG | Disruption of *Mst1* |
| DMst1-5-2 | | | GGGGACTGCTTTTTTGTACAAACTTGTTCGATATCCGGTGATTG |  |
| DMst1-3-1 | | | GGGGACAACTTTGTATAGAAAAGTTGTTAGGTCGACAAGATGATG |  |
| DMst1-3-2 | | | GGGGACAACTTTGTATAATAAAGTTGTTCGGACTGGAGATGAAG |  |
| DMst1-CF1 | | | TGAAGTCATCCGTCTTC | Confirming the disruption of *Mst1* |
| DMst1-CF2 | | | TGATGCTGGTGATGATG |  |
| DdMst1-5-1 | | | GGTCTAGAATAGCGTGTCGAGTACG | Double disruption of *Mst1* |
| DdMst1-5-2 | | | GGAGATCTGCTCGTCAGACAATGGA |  |
| DdMst1-3-1 | | | GGTCTAGAATCAAGGAGTGCAGAGG |  |
| DdMst1-3-2 | | | GGTCTAGACGAGTTTGATGCTGGTG |  |
| DdMst1-CF1 | | | GGAGCATGTATAGAGCT | Confirming the double disruption of *Mst1* |
| DdMst1-CF2 | | | TGTTAATGTTCGGAGCG |  |
| DMrt CF-1 | | | ATCGTCTGCGTCACATGG | Confirm the disruption of *Mrt* |
| DMrt CF-2 | | | GACATGTATGTATTTATG |  |
| Bar-up | | | CGCCTGGACGACTAAACC | Confirming gene deletion using *Bar* gene as a selection marker |
| Bar-down | | | TCAGCCTGCCGGTACCGC |  |
| Sur-up | | | ATCGTGGAGTCATGTTTG | Confirming gene deletion with *Sur* gene as a selection marker |
| Sur-down | | | CCAGTAAGTAATATATCC |  |
| C-ΔMst1-F | GGTCTAGACCTGATCCTGTAACAAC | | | Confirming complementation of *∆Mst1* |
| C-ΔMst1-R | | GGGAATTCTATTACCCTCTGCACTC | |  |
| *act*-F | | | TCCTGACGGTCAGGTCATC | Reference gene for  qRT-PCR |
| *act*-R | | | CACCAGACATGACGATGTTG |  |
| *gpd*-F | | | GTCGTCATCTCTGCTCCCTC | Reference gene for  qRT-PCR |
| *gpd*-R | | | CAATGGTGAACTTGTCGTGG |  |
| Mst1-RT-F | | | CACACCCACCCTGATCCTTC | qRT-PCR for Mst1 |
| Mst1-RT-R | | | AAAGACCGCCCCAGATGAAG |  |
| pGAD-st1-F | | GGAAGCTTATGTCTGGCGGTCTCGAG | | Heterologous expression Mst1 of yeast |
| pGAD-st1-R | | GGAAGCTTCTATACATGCTCCGCAGC | |  |
| ADH1-pro | | | AATGAGCAACGGTATACG | Confirming heterologous expression Mst1 of yeast |
| ADH1-ter | | | GTCAACAACGTATCTACC |  |
| ORF-5 | | | ATGTCTGGCGGTCTCGAG | Confirming the complementation of *∆Mst1* |
| ORF-3 | | | CTATACATGCTCCGCAGC |  |
